# DNA methylation profiling improves routine diagnosis of paediatric central nervous system tumours: A prospective population‐based study

**DOI:** 10.1111/nan.12838

**Published:** 2022-08-03

**Authors:** Elizabeth Schepke, Maja Löfgren, Torsten Pietsch, Thomas Olsson Bontell, Teresia Kling, Anna Wenger, Sandra Ferreyra Vega, Anna Danielsson, Sandor Dosa, Stefan Holm, Anders Öberg, Per Nyman, Marie Eliasson‐Hofvander, Per‐Erik Sandström, Stefan M. Pfister, Birgitta Lannering, Magnus Sabel, Helena Carén

**Affiliations:** ^1^ Childhood Cancer Centre, Queen Silvia Children's Hospital Sahlgrenska University Hospital Gothenburg Sweden; ^2^ Sahlgrenska Centre for Cancer Research, Department of Laboratory Medicine, Institute of Biomedicine, Sahlgrenska Academy University of Gothenburg Gothenburg Sweden; ^3^ Department of Neuropathology, DGNN Brain Tumour Reference Centre University of Bonn Medical Centre Bonn Germany; ^4^ Department of Clinical Pathology and Cytology Sahlgrenska University Hospital Gothenburg Sweden; ^5^ Department of Physiology, Institute of Neuroscience and Physiology, Sahlgrenska Academy University of Gothenburg Gothenburg Sweden; ^6^ Department of Clinical Neuroscience, Institute of Neuroscience and Physiology, Sahlgrenska Academy University of Gothenburg Sweden; ^7^ Department of Paediatrics Karolinska University Hospital Stockholm Sweden; ^8^ Department of Women's and Children's Health Uppsala University Uppsala Sweden; ^9^ Department of Paediatrics Linköping University Linköping Sweden; ^10^ Department of Paediatric Oncology and Haematology, Lund University Skane University Hospital Lund Sweden; ^11^ Department of Paediatrics Umeå University Umeå Sweden; ^12^ Department of Paediatric Haematology and Oncology Heidelberg University Hospital Heidelberg Germany; ^13^ Division of Paediatric Neuro‐oncology German Cancer Research Centre (DKFZ), Heidelberg Germany; ^14^ Department of Paediatrics, Institute of Clinical Sciences, Sahlgrenska Academy University of Gothenburg Gothenburg Sweden

**Keywords:** diagnostics, DNA methylation profiling, molecular classification, paediatric brain tumours

## Abstract

**Aims:**

Paediatric brain tumours are rare, and establishing a precise diagnosis can be challenging. Analysis of DNA methylation profiles has been shown to be a reliable method to classify central nervous system (CNS) tumours with high accuracy. We aimed to prospectively analyse CNS tumours diagnosed in Sweden, to assess the clinical impact of adding DNA methylation‐based classification to standard paediatric brain tumour diagnostics in an unselected cohort.

**Methods:**

All CNS tumours diagnosed in children (0–18 years) during 2017–2020 were eligible for inclusion provided sufficient tumour material was available. Tumours were analysed using genome‐wide DNA methylation profiling and classified by the MNP brain tumour classifier. The initial histopathological diagnosis was compared with the DNA methylation‐based classification. For incongruent results, a blinded re‐evaluation was performed by an experienced neuropathologist.

**Results:**

Two hundred forty tumours with a histopathology‐based diagnosis were profiled. A high‐confidence methylation score of 0.84 or more was reached in 78% of the cases. In 69%, the histopathological diagnosis was confirmed, and for some of these also refined, 6% were incongruent, and the re‐evaluation favoured the methylation‐based classification. In the remaining 3% of cases, the methylation class was non‐contributory.

The change in diagnosis would have had a direct impact on the clinical management in 5% of all patients.

**Conclusions:**

Integrating DNA methylation‐based tumour classification into routine clinical analysis improves diagnostics and provides molecular information that is important for treatment decisions. The results from methylation profiling should be interpreted in the context of clinical and histopathological information.

## Graphical abstract

In this population‐based study, a successful diagnostic molecular classification using DNA methylation was achieved in 78% of the cohort. Six per cent of the initial diagnoses were on re‐evaluation changed by an experienced neuropathologist favouring the methylation‐based diagnosis. The change in diagnosis would have influenced the management of the patients in 5% of the cohort.

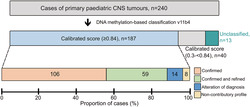

**Key points**

- DNA methylation has a key role (up‐front) in a population‐based diagnostic setting for paediatric CNS tumours.
- Methylation‐based tumour classification enhances the diagnostic information, helps identify rare entities and allows for a change in the management of patients.
- The data demonstrate that also tumours with low tumour cell content can be well classified.

## INTRODUCTION

Paediatric central nervous system (CNS) tumours are rare and show great heterogeneity, which makes the diagnosis challenging. The classification is based on histopathologic and molecular criteria as outlined by the World Health Organisation (WHO) as well as the location of the tumour [1]. Today, more than 100 different CNS tumour entities with varying grade of malignancy are distinguished. Determining the neuropathological diagnosis can be difficult, and previous studies have reported high inter‐observer variability in the diagnostics for some tumour entities [2, 3].

CNS tumours are the second most common group of tumours in children, after leukaemia/lymphoma, but account for the majority of cancer‐related deaths [4]. These tumours are currently treated with surgery, often followed by chemo‐ and/or radiotherapy and in selected cases with targeted therapy. In addition to the relatively high overall mortality [5], childhood brain tumour survivors often suffer from serious side effects, both short‐term and long‐term, with considerable risks of neurological, endocrine and cognitive health problems [6, 7, 8]. An accurate diagnosis of paediatric tumours is crucial for the choice of treatment and to achieve the optimal balance between likelihood of long‐term cure and avoidance of excessive treatment.

During the last decade, DNA methylation profiling has been shown to be a reliable and robust method to classify paediatric CNS tumours [9, 10]. The technique is reproducible for analysing fresh‐frozen tumour samples as well as formalin‐fixed paraffin‐embedded (FFPE) tumour samples [11]. DNA methylation profiling is recognised as an important tool to stratify paediatric brain tumour patients into clinically relevant subgroups [12, 13, 14, 15] and to better predict prognosis and response to treatment [16, 17]. Recent studies showed that using conventional histopathological and molecular diagnostics in combination with DNA methylation profiling can refine tumour diagnoses and sometimes lead to a change of the final diagnosis [18, 19, 20].

The impact of using DNA methylation analysis in the diagnostics of paediatric brain tumours has been demonstrated in several studies. However, some series were enriched for diagnostically challenging cases and high grade tumours [18, 21] and several retrospective analyses lacked a diagnostic re‐analysis by state of the art neuropathologic evaluation. In this analysis, we fully investigated an unbiased series of unselected cases. Therefore, in this study, we aimed to investigate the impact of performing DNA methylation profiling in routine diagnostics, for all children diagnosed with a CNS tumour in Sweden during a four‐year period.

## MATERIAL AND METHODS

### Patients and samples

All paediatric patients (<18 years old) diagnosed with a CNS tumour at one of the six paediatric neurosurgery‐oncology centres in Sweden between 1 January 2017 and 31December 2020 were eligible for the study, provided sufficient FFPE tumour material was available for DNA methylation analysis. Histopathological reports including immunohistochemistry and molecular analyses were performed at the six pathology departments involved in diagnosing paediatric CNS tumours in Sweden. DNA extraction and DNA methylation array analysis were centralised to Carén lab at Sahlgrenska Centre for Cancer Research in Gothenburg.

Clinical data were obtained from the Swedish Childhood Cancer Registry. For all patients included in the study, complete histopathological and molecular standard diagnostics was performed prior to the methylation analysis.

Additional tumour samples from 32 patients with tumour relapse, diagnosed during the study period, were analysed with methylation array. The primary operation in these cases had been performed years earlier. Tumour samples from the primary operations were also collected and analysed in the same order as above. In total, 66 samples (initial diagnosis and relapse and for two cases also a second relapse) were collected.

### Tumour cell content

The proportion of tumour cells in each sample was estimated by two neuropathologists (TOB, SD). The assessment was based on haematoxylin and eosin‐stained slides, taken before and after slicing the FFPE block used for the array analysis. We defined a high tumour cell content as ≥70% tumour cells. All tumour samples were analysed with the methylation array regardless of the tumour cell content.

### DNA extraction and quantification

Tumour DNA was extracted from sections of FFPE tumour blocks and extracted with QIAamp® DNA FFPE kit (Qiagen, Hilden, Germany) following the protocol provided by the manufacturer, with an extra proteinase K digestion step as previously described [22] or by using the Maxwell® FFPE Plus DNA Kit with a Maxwell® RSC (Promega, Wisconsin, USA). The extracted DNA was quantified using Qubit® Fluorometer Invitrogen (ThermoFisher, Waltham, Massachusetts, USA).

### Bisulfite conversion of DNA, restoration and array processing

Approximately 500 ng of extracted DNA was bisulfite‐converted with EZ DNA methylation kit (Zymo Research, Irvine, CA, USA) and restored with the Infinium HD FFPE Restore Kit (Illumina, San Diego, CA, USA) according to the instructions supplied by the manufacturer. The Infinium MethylationEPIC Bead‐Chip array (Illumina) was used to generate genome‐wide DNA methylation profiles.

### Methylation‐based classification

For methylation‐based tumour classification, raw data (idat files) were uploaded to the openly available DNA methylation‐based classifier, MNP version 11b4 (www.molecularneuropathology.org). This resulted in a report, indicating the best predicted match of a methylation‐based tumour diagnosis and a corresponding calibrated score (CS), calculated using an algorithm from the German Cancer Research Centre (DKFZ) [12] ranging from 0–1, and a chromosomal copy number variation (CNV) plot. The brain tumour classifier v11b4 comprises 82 CNS tumour methylation classes and nine control tissue methylation classes [12]. In line with previous publications [19, 21] and as suggested by Capper et al. for clinical settings [15], a CS ≥0.84 indicated successful classification.

The brain tumour classifier version 11b4 was recently updated to MNP version 12.5. www.molecularpathology.org/mnp (unpublished). Therefore, all 240 tumour samples were re‐analysed using this new version, which includes 184 molecular tumour classes, subclasses and control tissue classes. We applied the same cut‐off for successful classification (CS ≥ 0.84), although the optimal cut‐off for this version has not been fully investigated.

Furthermore, all non‐WNT/non‐SHH medulloblastomas were also re‐analysed in the specific medulloblastoma classifier: medulloblastoma classifier group 3/4 version 1.0, which classified these medulloblastomas further into the subtypes *I–VIII*, www.molecularpathology.org/mnp.

### Diagnostic impact

In order to establish what potential impact, the methylation‐based classification would have had on the final diagnosis if used up‐front, the original histopathology reports were reviewed. This assessment was done by two authors (TOB, ES) independently of the reporting neuropathologists. When the CS was ≥0.84, the impact was categorised as one of the following: *(I) confirmed the diagnosis*, that is, the methylation‐based classification and the histopathological diagnosis were in agreement; *(II) confirmed and refined the diagnosis*, that is, providing additional molecular subtyping information to standard diagnostics; *(III) altered the initial diagnosis and would have changed the final diagnosis* if the methylation‐based classification had been included in real time diagnostics; or *(IV) considered non‐contributing or misleading*.

When the CS was <0.84, it was considered as *(V) a lower confidence score* when the CS was 0.3–0.83 or *(VI) unclassified* when the CS was <0.3.

### Neuropathological re‐evaluation

All cases with a high‐confidence CS ≥0.84 that differed from the diagnosis in the original neuropathological report, and cases with a lower confidence score (<0.84) but with a high tumour cell content, were re‐evaluated by an experienced neuropathologist (TP). These samples were re‐evaluated and classified according to WHO 2016 using immunohistochemical and molecular analyses [1]. The reviewing neuropathologist was blinded for the original histopathological reports as well as for the results from the methylation profiling.

### Statistics

For statistical analyses, the statistical software R was used [23]. Comparisons of CSs and tumour cell content between groups were performed using Welch two sample *t* test. The significance level was set to *p* = 0.05.

### Ethics

The study was approved by the regional ethics committee in Gothenburg, Sweden (Dnr 604‐12, T1162‐16). Informed consent was obtained from the guardians.

## RESULTS

### Patients' characteristics

**FIGURE 1:**
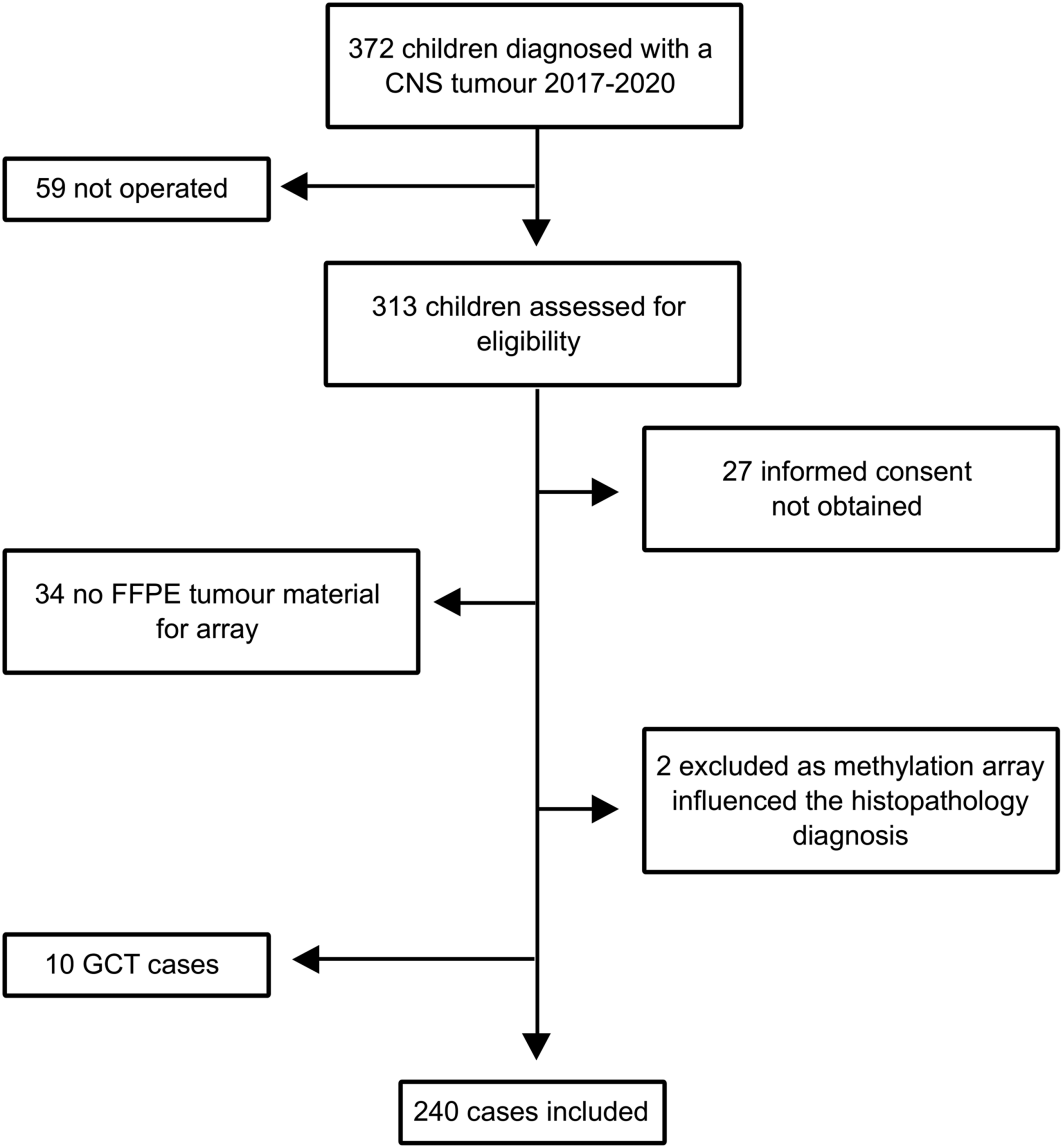
Cohort description. FFPE = formalin‐fixed paraffin‐embedded, GCT = germ cell tumour.

In all, 372 paediatric patients (0–18 years old) were diagnosed with a CNS tumour in Sweden during 2017–2020. In 313 cases, a tissue‐based diagnosis was obtained. Patients with insufficient FFPE tumour tissue for methylation analysis or when informed consent was not obtained were not included, Figure 1. Two patients were excluded from the analysis of the diagnostic effect since a DNA methylation array had already been performed as part of the clinical diagnostic work‐up, influencing the original diagnosis. Ten cases with germ cell tumours (GCTs) were excluded as this tumour class is not included in the classifier version 11b4.

A total of 240 eligible patients remained with tumour tissue available for DNA methylation array analysis. There were 125 male and 115 female patients. The mean age at diagnosis was 8 years (range: 0–17.9, median 7.8). In total, 224 children (93%) had open surgery, and 16 underwent a biopsy. Fifty per cent of the tumours were infratentorial. The distribution of tumours and clinical characteristics are listed in Table 1.

**TABLE 1:** Clinical and tumour characteristics for 240 children operated for a primary CNS tumour 2017–2020^a^

	*N* (%)
Gender	
Male	125 (52)
Female	115 (48)
Ratio male/female	1.1
Age at diagnosis, mean (years)	8.4
Tumour location	
Cerebrum	90 (38)
Chiasma/hypothalamus	5 (2)
Sellar/suprasellar	10 (4)
Cerebellum	108 (45)
Brainstem	13 (5)
Spine	14 (6)
Type of surgery	
Resection	224 (93)
Biopsy	16 (7)
Tumour types based on histopathology (WHO 2016)	
Low‐grade glioma and glioneuronal tumours	114 (48)
High‐grade astrocytomas	18 (7)
Medulloblastoma	43 (18)
Ependymoma	21 (9)
Craniopharyngioma	10 (4)
Choroid plexus tumours	6 (2)
Atypical teratoid rhabdoid tumour	4 (2)
Others	24 (10)

Abbreviations: CNS, central nervous system; WHO, World Health Organisation.

^a^ Data are presented as number of patients (*N*) and percentage (%).

### DNA methylation‐based classification

**FIGURE 2:**
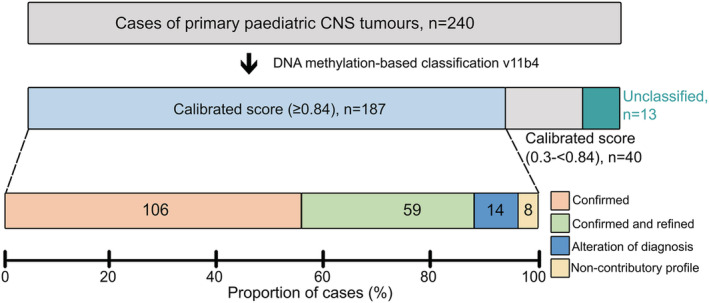
Result of DNA methylation classification of paediatric central nervous system (CNS) tumours. Of the 240 profiled cases, 187 tumours (78%) were classified with a high calibration score ≥0.84. The diagnostic impact of methylation profiling on the initial histopathological diagnosis was categorised into Diagnostic *(I)* confirmation (light pink); *(II)* confirmed and refined diagnosis (light green); *(III)* alteration of diagnosis (blue); *(IV)* non‐contributory profile (yellow); *(V)* low calibrated scores (grey); and *(VI)* unclassified (turquoise).

From the 240 primary tumours, the classifier tool (MNP version11b.4) assigned 187 cases (78%) to a specific DNA methylation class with a CS of 0.84 or higher, Figure 2. In 40 cases (17%), methylation classification produced lower confidence CSs, between 0.3 and <0.84. For cases with a class‐prediction CS < 0.30, a methylation class could not be predicted. This result, here referred to as ‘unclassified’ cases, was observed in 5% (13/240) of cases and will be discussed below. All samples had a probe failure rate less than 1.5%, with 99% of the samples less than 1%.

### Diagnostic impact of methylation profiling

#### I Confirmation of diagnosis and II confirmation and refinement of diagnosis (CS ≥ 0.84)

**FIGURE 3:**
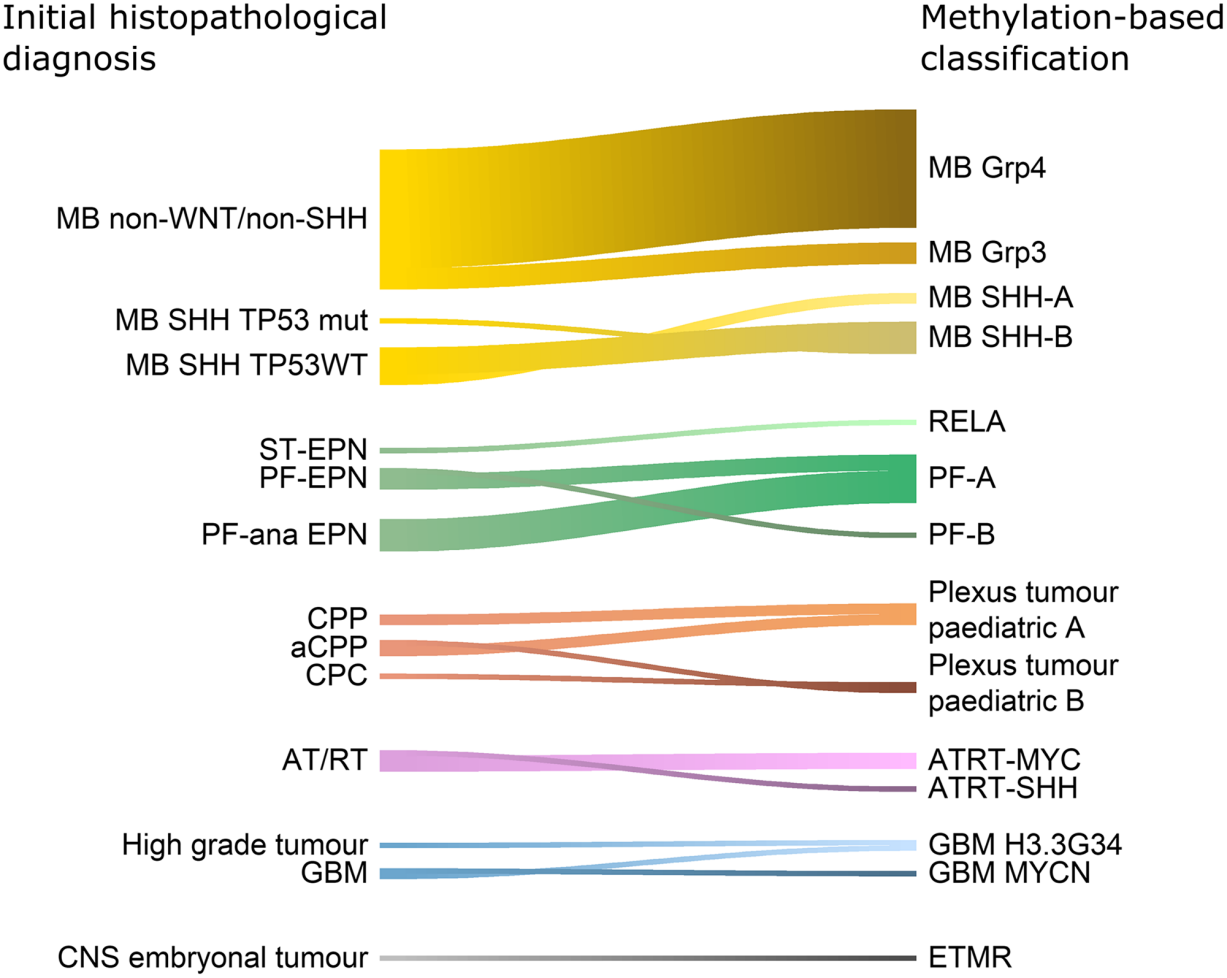
Refinement of diagnosis by methylation profiling in 59 tumours with varying initial histological diagnoses (WHO 2016) (left) and corresponding methylation classes (right). MB, medulloblastoma (*n* = 34); MB Grp4, subgroup 4 (*n* = 22); MB Grp3, subgroup 3 (*n* = 4); MB‐SHH‐A, medulloblastoma sonic hedgehog child and adolescent group (*n* = 2); MB‐SHH‐B, medulloblastoma sonic hedgehog infant group (*n* = 6); ST‐EPN, supratentorial ependymoma (*n* = 1); PF‐EPN, posterior fossa ependymoma (*n* = 4); PF‐ana EPN, anaplastic ependymoma in posterior fossa (*n* = 6); RELA, RELA‐fusion positive ependymoma (*n* = 1); PF‐A, posterior fossa ependymoma subgroup A (*n* = 9); PF‐B, posterior fossa ependymoma subgroup B (*n* = 1); CPP, choroid plexus papilloma (*n* = 2); aCPP, atypical choroid plexus papilloma (*n* = 3); CPC, choroid plexus carcinoma (*n* = 1); AT/RT, atypical teratoid/rhabdoid tumour (*n* = 4); AT/RT‐MYC, subclass MYC (*n* = 3); AT/RT‐SHH, subclass SHH (*n* = 1); GBM, glioblastoma (*n* = 2); GBM H3.3 G34, glioblastoma with H3F3A G34 mutation (*n* = 1); GBM MYCN, glioblastoma IDH wildtype subclass MYCN (*n* = 1); ETMR, embryonal tumour with multi‐layered rosettes (*n* = 1)

In 165/240 cases (69%), the predicted methylation class confirmed the initial neuropathological diagnosis. In 59 of these cases (25%, 59/240), the initial neuropathological diagnosis was not only confirmed but also refined by the added information gained by DNA methylation, for example, providing molecular subgrouping data not available with standard diagnostics. This refinement of diagnosis mainly occurred in medulloblastomas (*n* = 34) where medulloblastoma subgroups could be established, ependymomas (*n* = 11) where RELA‐fusion or posterior fossa type A or B could be specified and choroid plexus tumours (*n* = 6) or AT/RT (*n* = 4) where methylation subclasses were identified, Figure 3.

#### III Alteration of diagnosis (CS ≥ 0.84)

For 14/240 cases (6%), the predicted diagnoses from the methylation‐based classification were incongruent with the original neuropathological diagnoses. Furthermore, the diagnoses suggested by the reference neuropathologist agreed with the methylation‐based classification, leading to the revised diagnoses shown in Table 2 and described in detail below for selected cases.

**TABLE 2:** Revised diagnoses based on histopathological re‐evaluation

Case	Initial histopathological diagnosis	MC	CS	Revised diagnosis
1	PA	DLGNT	0.98	DLGNT
2	PA	DLGNT	0.99	DLGNT
3	PA	RGNT	0.99	RGNT
4	Pilocytic/pilomyxoid astrocytoma	Low‐grade glioma MYB/MYBL1	0.99	Angiocentric glioma
5	Low‐grade glial tumour, NOS	(ana) PXA	0.98	PXA
6	Low‐grade tumour, NOS	PA	0.92	PA
7	Glial or glioneuronal tumour, uncertain grade	PA	0.99	PA
8	SEGA	(ana) PXA	0.98	Epithelioid GBM with relation to PXA
9	Undifferentiated tumour, (II)	Infantile hemispheric glioma	0.96	Infantile hemispheric glioma
10	Malignant undifferentiated tumour (IV)	(ana) PXA	0.99	Epithelioid glioblastoma
11	Malignant high‐grade tumour (IV)	CNS NB with FOX R2 activation	0.99	CNS NB with FOX R2 activation
12	CNS neuroblastoma	MB, Grp 3	0.93	Metastatic medulloblastoma without primary cerebellar tumour
13	DIA	(ana) PXA	0.99	PXA
14	Ganglioglioma	(ana) PXA	0.95	PXA

Abbreviations: (ana) PXA, (anaplastic) pleomorphic xanthoastrocytoma; CNS NB, CNS neuroblastoma; CS, calibrated score; DIA, desmoplastic infantile astrocytoma and ganglioglioma; DLGNT, diffuse leptomeningeal glioneuronal tumour; GBM, glioblastoma; MC, methylation classification; PA, pilocytic astrocytoma; PXA, pleomorphic xanthoastrocytoma; RGNT, rosette forming glioneuronal tumour; SEGA, subependymal giant cell astrocytoma.

*Cases 1 and 2* were initially diagnosed as pilocytic astrocytomas (PA) but classified as diffuse leptomeningeal glioneuronal tumours (DLGNTs) by the classifier‐tool. These tumours had clinicopathological features of DLGNT [24]; that is, leptomeningeal enhancement of the spinal cord or loss of chromosome arm 1p visualised on the CNV plot.

*Case 9* was originally an undifferentiated tumour WHO grade II by histopathological diagnosis but with a revised diagnosis of infantile hemispheric glioma. Following further investigations triggered by the methylation classification result, a *ROS1* fusion was detected.

**FIGURE 4:**
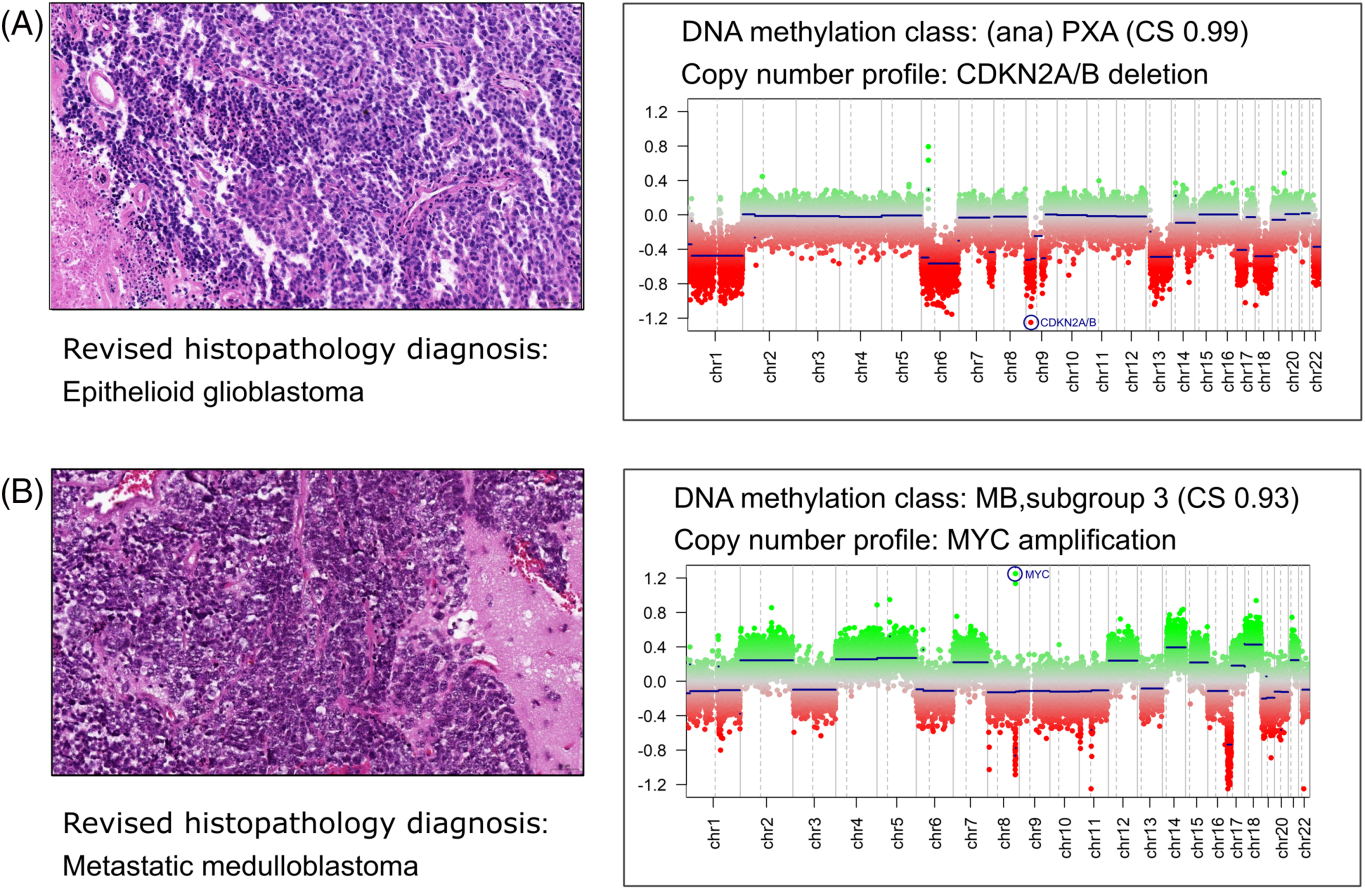
Histopathology and copy number variation (CNV) plots for two cases with revised diagnosis. (A) Case 10 (Table 2) a revised histopathology diagnosis of epithelioid glioblastoma (haematoxylin and eosin stain [H&E]) and the methylation class and CNV plot corresponding to an (anaplastic) pleomorphic xanthoastrocytoma (PXA). (B) Case 12 (Table 2) with the initial diagnosis favouring CNS neuroblastoma with the DNA methylation class medulloblastoma, group 3 and CNV plot with MYC amplification. The revised histopathology diagnosis was a metastatic medulloblastoma.

*Case 10* with descriptive histopathological diagnosis of malignant undifferentiated tumour was classified by methylation profiling as (anaplastic) pleomorphic xanthoastrocytoma (PXA). CNV analysis showed several chromosomal alterations, including homozygous deletion of *CDKN2A/B*. No *BRAF* V600E mutation was detected. On re‐evaluation, this tumour was diagnosed as an epithelioid glioblastoma (GBM), Figures 4A and S1a. The highly proliferative tumour did not present the essential criteria for the diagnosis of PXA; it lacked xanthomatous/lipidized cells and eosinophilic granular bodies. It also lacked reticulin deposition and typical CD34 expression. Epithelioid GBMs show similar methylation profiles as PXAs [15, 25]. Further diagnostic exploration revealed a *TRIM24::NTRK2* gene fusion.

*Case 12* was diagnosed with a frontal tumour and a histological description of a highly cellular, small blue cell‐like neoplasm, favoured as a CNS neuroblastoma. DNA methylation profiling demonstrated a medulloblastoma, subgroup 3 and the CNV plot showed *MYC* amplification, Figures 4B and S1b. The re‐evaluation favoured a metastatic medulloblastoma with an undetected primary tumour of the cerebellum. Such cases are described by Capper et al. [15] and may represent a rare, not currently defined, tumour entity or a very rare presentation of a ‘primary leptomeningeal medulloblastoma’, that is, a medulloblastoma without a detectable macroscopic tumour in the posterior fossa [26].

*Case 14* had an initial diagnosis of a (temporal) ganglioglioma but was classified by methylation as a PXA. The CNV plot showed deletion of *CDKN2A/B*. A *BRAF* V600E mutation was found. Pathology review favoured a diagnosis of PXA.

**FIGURE 5:**
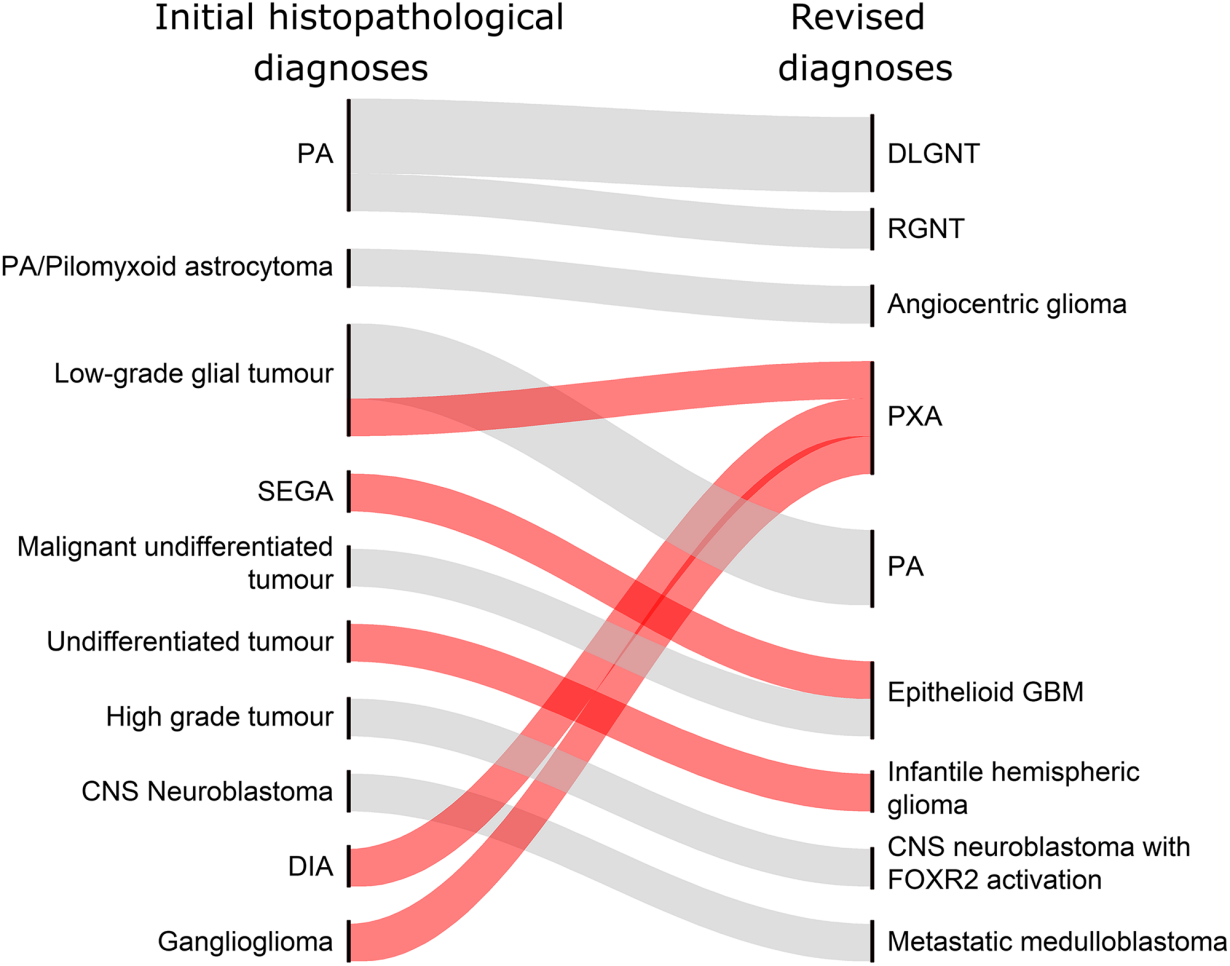
Change of World Health Organisation (WHO) grading for the 14 revised cases. Initial histopathological diagnoses (left) and the revised diagnoses (right) after blinded re‐evaluation. PA, pilocytic astrocytoma; DLGNT, diffuse leptomeningeal glioneuronal tumour; RGNT, rosette forming glioneuronal tumour; PXA, pleomorphic xanthoastrocytoma; SEGA, subependymal giant cell astrocytoma; GBM, glioblastoma; DIA, desmoplastic infantile astrocytoma and ganglioglioma. WHO grading changes are shown in red (escalated). Grey represents unchanged grading.

In five cases the change in diagnosis resulted in an escalation of the WHO grade, Figure 5, whereas no tumour was downgraded.

Two senior paediatric neuro‐oncologists (BL, MS) assessed that the management of the patients would have changed in 11 of these 14 cases, that is, 5% of the total cohort. This included changes in follow‐up (*n* = 5), treatment plans (*n* = 4) or potential use of targeted therapy (*n* = 2).

#### IV Non‐contributing or potentially misleading (CS ≥ 0.84)

In 8/240 cases (3%), the methylation analysis predicted brain control tissue classes (cerebellar hemisphere or reactive tumour microenvironment), which obviously did not match with the histopathology. Furthermore, the pre‐operative MRIs of all these patients, showed a tumour. However, all these samples had a low tumour cell content (15–50%), and on re‐evaluation, there were a lot of cerebellar granular cells and signs of reactive tissue in some of the samples. In all these eight cases, when seen in relation to radiological and pathological data, as well as to the low tumour cell content, we do not consider the methylation‐based classification of brain control tissue as misleading but rather non‐contributory.

#### V Evaluation of cases with lower CSs (CS 0.3–<0.84)

Forty patients had lower (non‐diagnostic) CSs (0.3–<0.84), Figure 2. In the majority (65%) of these cases, a low tumour cell content was seen. Despite this, in 19/40 cases, the suggested methylation‐based classifications were concordant with the initial histopathological diagnoses. Nine additional samples were predicted as control brain tissue. In the other 12 discrepant cases, there were tumours with a relatively high tumour cell content (50–80%). Two examples: One case was initially diagnosed as DLGNT but was predicted to be a PA by methylation (CS 0.82). The CNV plot did not show the 1p deletion typical of DLGNT, and the magnetic resonance imaging (MRI) had no typical features of DLGNT. The second case was a spinal PA, predicted to be a DLGNT with methylation classification (CS 0.77). The tumour had 1p deletion, and the MRI showed leptomeningeal enhancement. On reassessment by the neuropathologist, both diagnoses changed in favour of the methylation classification.

#### VI Unclassified cases

The remaining 13/240 cases (5%) could not be classified by the MNP version 11.b4 with a score >0.3. Six were low‐grade tumours or craniopharyngiomas with a low tumour cell content (20–40%), and seven were unclassified tumours with a high tumour cell content, which were re‐evaluated. Two had a revised histopathological diagnosis; one as a tanycytic ependymoma, a diagnosis not included in the classifier v11.b4; and the other as a supratentorial ependymoma where the array‐generated CNV plot showed a chromosome 22 deletion. The latter case was an ependymoma with a chromosome 22 deletion likely not recognised by the classifier but described by Zschernack et al. [27]. When reviewed, the other five unclassified tumours were considered to be unusual and undifferentiated tumours, most likely representing rare novel entities.

### Tumour types and CS

**FIGURE 6:**
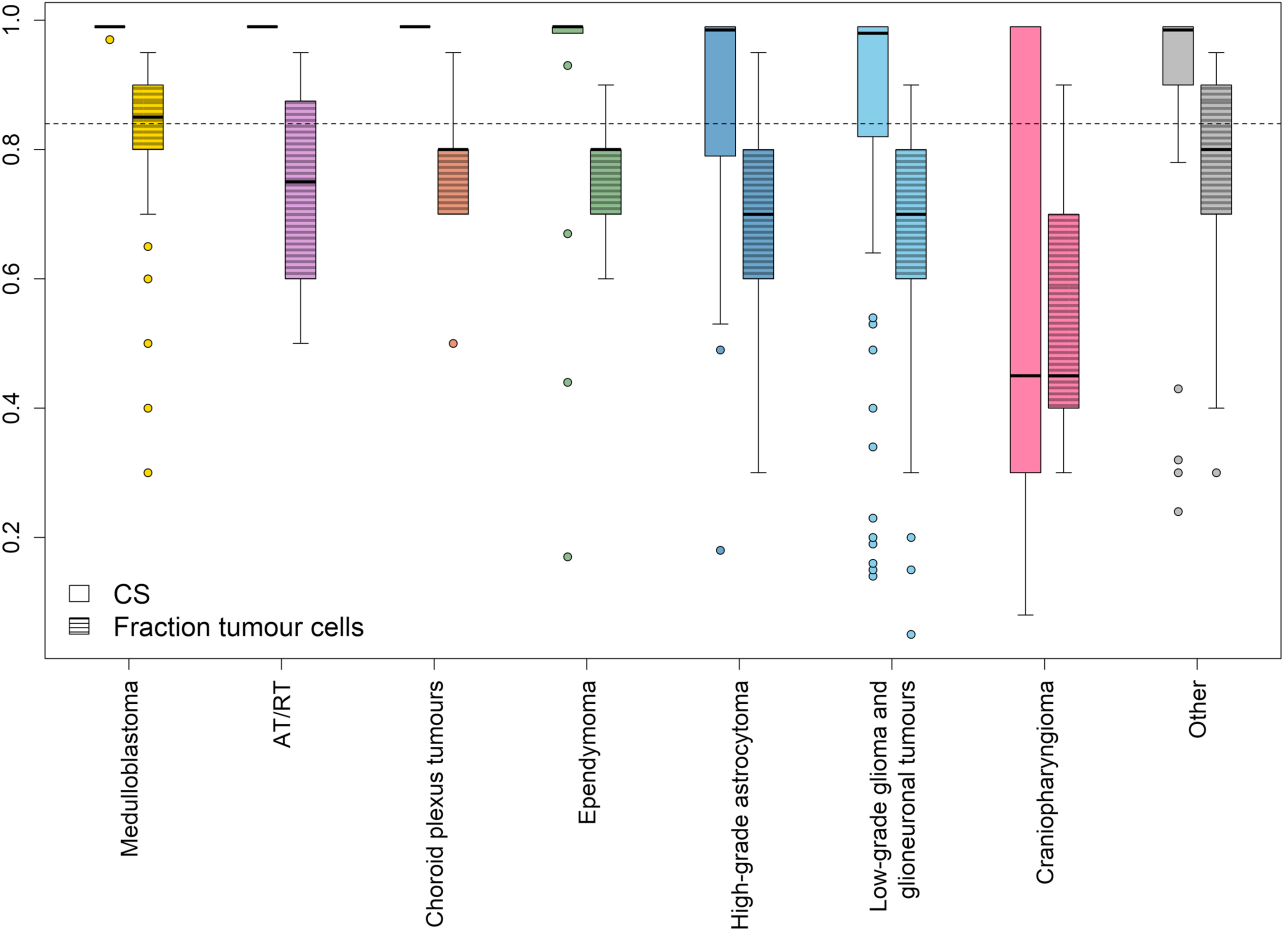
For each tumour type, boxplots are shown for DNA methylation calibrated score (CS) and for proportion of tumour cells in samples. The dotted line shows the 0.84 CS threshold. Upper and lower hinges of the box represent the 75th percentile and 25th percentile, respectively; whiskers indicate the highest and lowest values that are not outliers; thick horizontal line within the box, median. Open circles represent outliers. The tumour cell content was estimated based on haematoxylin and eosin‐stained slides.

We also investigated how the class prediction scores varied in relation to the different tumour types, based on the initial neuropathological diagnoses, Figure 6. The most confidently classified tumours were medulloblastomas, in which 100% of the 43 profiled cases received confident scores. AT/RTs and choroid plexus tumours were also all accurately classified with a CS ≥ 0.84. Ependymomas were well classified, with 18 of the 21 cases (86%) confidently classified. Notably, all RELA‐altered ependymomas were successfully classified by the methylation‐based classification. Eighteen patients were diagnosed with a high‐grade astrocytoma (grades III and IV), and 13 (72%) of these were confidently classified with the largest number of classified cases in H3.3 G34‐mutated GBMs (75%). Three out of six diffuse midline gliomas with a histone H3K27 mutation were classified with lower CSs (0.49–0.79). These were located in the pons and tissue obtained via biopsies. For low‐grade gliomas and glioneuronal tumours, a CS of ≥0.84 was noted in 71% of all 114 cases, compared with our total cohort's average of 78%. Notably, the craniopharyngiomas showed the lowest CSs.

### Tumour cell content and CS

Overall, 69% of the tumour samples were estimated to have a high tumour cell content, that is, 70% or more. The proportion of samples with a successful CS (≥0.84) was higher with increasing tumour cell content. However, when looking at the tumour cell counts in relation to the different tumour types, several cases had a high confidence class prediction score despite a low tumour cell content, Figure 6. Some medulloblastoma samples had a relatively low tumour cell content but were still accurately classified. For low‐grade gliomas and glioneuronal tumours, almost 55% of the samples had a low tumour cell content, which probably explains why some of these were not assigned to a specific tumour class. Craniopharyngiomas had the lowest number of neoplastic cells (mean 55%) compared with other tumours (*p* = 0.043) as well as the lowest CSs (mean 0.53) (*p* = 0.009), likely related to tumour cell content rather than the tumour type itself [28].

### Methylation‐based re‐analysis on all 240 tumour samples, using MNP version 12.5

To compare the newer MNP Classifier version 12.5 (unpublished) to version 11b4, we re‐analysed the methylation data for all the 240 samples with version 12.5, using the same cut‐off for successful classification (CS ≥ 0.84). A greater proportion of samples, 87% (208/240), were assigned to a specific methylation class with version 12.5 (Figure S2), compared with 78% with version 11b4. In 185/240 cases (77%), the predicted methylation‐based classification confirmed or refined the histopathological diagnosis, compared with 69% with version 11b4. The newer version of the classifier successfully classified 21 additional tumours that had not been confidently classified with the older version. The predicted diagnoses were in congruence with the histopathological diagnoses and were mainly low grade astrocytic or glioneuronal tumours that had received low to intermediate classification scores with version 11b4, as well as some (5/13) previously *unclassified cases* (CS < 0.3). A few higher‐grade tumours were also among the additional tumours successfully classified with version 12.5, including a GBM, a malignant astrocytoma and an ependymoma.

In 18/240 cases (8%), the predicted diagnoses using version 12.5 were incongruent with the original histopathological diagnoses including the cases with altered diagnosis with v11b4 (discussed previously). The remaining cases classified by the newer version involved tumours that had been very difficult to specify by the neuropathologists. Here, the methylation classifier suggested a new entity, the methylation class *neuroepithelial tumour with BCOR alteration*, suggesting the (WHO 2021) diagnosis of a *CNS tumour with BCOR internal tandem duplication*, as well as two supratentorial ependymomas and one GBM of paediatric type. In 5/240 cases (2%), the methylation analysis predicted (CS ≥ 0.84) brain control tissue classes. All these samples had a low tumour cell content (20–50%).

For 23/240 samples (10%), the class‐prediction score was <0.84 but above 0.3 (median 0.63). Still, in almost half of these cases, the suggested methylation class was in concordance with the initial histopathological diagnoses despite the lower confidence score. The other cases were non‐concordant or indicated brain control tissue (9/23). The majority of these samples had a tumour cell content <40%. In 9/240 cases, methylation profiles could not be classified by the MNP version 12.5 with a score of >0.3 (unclassified cases).

No further analyses were performed in these cases as this was beyond the scope of this study.

### Methylation‐based analysis using the specific medulloblastoma classifier group 3/4 1.0

The 26 non‐WNT/non‐SHH medulloblastomas were re‐analysed with the specific medulloblastoma classifier for group 3 or 4 medulloblastomas. All tumours retained their predicted genetic subgroup between classifier versions 11b4 and 12.5 as well as the medulloblastomas classifier. Four cases classified as subgroup 3; the remaining 22 were classified as subgroup 4. In two samples, the subtypes changed from subtype VIII in the classifier v12.5 to subtype VI in the medulloblastoma classifier (sample‐id 8 and 10) (Table S1).

### Relapses

We compared the predicted methylation class in the 66 paired tumour samples (primary and relapse). The suggested methylation class remained the same in both primary and relapsed tumour in 52/66 samples (79%). Several of the relapsed cases were tumours with unusual histology that had been difficult to classify and had been subjected to second opinion. For these cases, the methylation classification (MNP v11b4) could not predict a confident methylation class, whereas the MNP Classifier version 12.5 in two cases predicted new tumour entities; one was classified as a neuroepithelial tumour with *PATZ1* fusion and the other one as a high grade neuroepithelial tumour with *PLAG*‐family amplification, which had relapsed three times despite intense treatment. Both cases were classified with high‐confidence scores (CS 0.97‐1) in paired samples.

## DISCUSSION

In this population‐based study, we evaluated the diagnostic outcome of combining DNA methylation analyses with standard histopathological evaluation, in a cohort of 240 consecutive paediatric patients diagnosed with CNS tumours in Sweden. With this setup, our collection of tumours mirrors the general occurrence of paediatric CNS tumours in Sweden [5]. Undoubtedly, DNA methylation analysis is a robust and non‐biased method that strengthens the diagnostic accuracy.

Successful (diagnostic) molecular classification by DNA methylation analysis was achieved in 78% of the cohort using the MNP classifier v11b4 [12]. Previous studies have shown classification rates of 49–72% [18, 19, 20, 21]. The higher classification rate in our cohort could be explained by the population‐based setup of our study, rather than the investigation of a mixed cohort of paediatric patients composed of diagnostically challenging cases or cases referred for second opinion as in previous studies.

In the majority of cases (69%), the histopathological diagnoses and the methylation‐based diagnoses were in agreement. In 25% of the whole cohort, the methylation profiling did not only confirm but also refined the initial diagnosis, for example, by giving a more precise subgroup. This diagnostic refinement is important from a prognostic point of view as different molecularly defined tumour types have a distinct clinical behaviour [16, 29] and increasingly also from a therapeutic viewpoint.

In this study, 6% of the initial diagnoses were changed when re‐evaluated by an experienced reference neuropathologist, who was unaware of the methylation profiling results. All diagnoses were changed in favour of the predicted methylation class showing that the integration of methylation analysis in routine clinical diagnostics improves the diagnostic accuracy. It also provides guidance for additional diagnostic testing. However, methylation‐based classification could not help in differentiate in tumour grade; for example, PXA cannot be differentiated from anaplastic PXA or epithelioid GBM by methylation analysis.

Obviously, diagnostic classification is important for correct assignment to a specific treatment, but the change in diagnosis does not always lead to a change in treatment. In our study, we estimated that the change in the diagnosis would have altered the management for 11 (5%) of the patients, that is, a change of treatment or a different follow up. This is of utmost importance in paediatric neurooncology and consistent with previous reports [18]. In our judgement, no methylation classification was misleading when seen in relation to clinical and radiological data.

When the classifier cannot find a class prediction with a high confidence score, the interpretation may be problematic. The lower the score, the higher the rate of misleading diagnosis [18]. In this study, 53/240 cases could not be assigned to a DNA methylation class with a CS of ≥0.84 using MNP v11b4. The reasons for scores below the cut‐off cannot always be determined with certainty, but several explanations are possible; for example, the amount of DNA was too low or of poor quality, the tumour cell content was too low or the actual tumour entity was not included in the classifier‐algorithm. A recent study [21] found that the tumour cell content was the factor most significantly associated with the classifier score when comparing the amount of DNA in the sample, the tumour cell content and the tumour type. However, a suggested methylation‐based classification with a CS <0.84 may give important molecular information, especially when the score is >0.5 [15].

Most previous studies have only included samples with a high tumour cell content, ≥70%, in order to increase the possibility of a match to a predicted class. As our study was population‐based, all samples were subjected to methylation profiling independently of the tumour cell content in the samples. Tumour cell content was determined by histology, as this is the standard procedure in clinical work, even though it is known to be inaccurate due to interobserver variability [30].

As in previous studies [15, 19], it is clear that the tumour cell content is important in order to achieve a confident class score. Previous studies have shown the benefit of performing methylation profiling mainly in high‐grade tumours or challenging cases [18, 19, 31]. However, this study shows that the possibility of a correct classification also depends on the tumour type itself. Thus, although low‐grade gliomas and glioneuronal tumours often showed a low tumour cell content, they were predicted with high confidence scores in 71% of cases compared with our total cohort's average of 78%. This was a higher percentage than expected in comparison with other reports [10, 11, 12, 13, 14, 15, 16, 17, 18]. Consequently, in our opinion, one should not refrain from performing the analysis based only on a low tumour cell count.

When examining the relapses with paired samples, more than 70% had a defined methylation class, which was unchanged between primary diagnosis and relapse, showing that the methylation profiling is robust despite treatment, probably because it reflects the cell of origin of the tumour [32]. The remaining samples from the relapses mainly consisted of cases that were difficult to classify and unusual cases.

When re‐analysing all tumour samples with the newer unpublished MNP brain tumour methylation classifier version 12.5, several more samples reached a high confidence prediction score independently of tumour cell content. Not only were more samples confidently assigned to a specific tumour class, confirming or even refining the diagnosis, but also were new entities identified and difficult cases solved. This shows that the DNA methylation classification is gradually evolving, and even more tumour types with new molecular alterations will be included in future versions. In the recent 2021 WHO classification of CNS tumours, several new tumour types and subtypes were established, and many demand an advanced level of molecular diagnostics [33]. DNA methylation provides an additional molecular layer in this respect.

To our knowledge, no population‐based study has been performed that has included all CNS tumours from an entire nation during a defined time‐period. Our study shows that DNA methylation analysis has an added value in the diagnostics of paediatric CNS tumours and in treatment decisions, if used as a complement to standard neuropathology. Using a newer version of the classifier, with more diagnoses included, several diagnoses changed, and new entities were identified. One important aspect when using DNA methylation in real time diagnostics is to keep the turnaround time as short as possible in order to integrate the classification result in the pathological diagnosis. A result from a methylation analysis could potentially be obtained within 10 days from operation [12]. In many countries, this requires a centralised analysis also for keeping the costs as low as possible. In one study [19], the use of methylation arrays was considered both cost‐effective and tissue‐saving for diagnostically difficult cases.

A limitation of the study is that microdissection was not used. In fact, histopathological evaluation prior to dissection for molecular diagnostics (including methylation‐based classification) is considered current standard of care for neuropathological diagnostic procedures. With dissection, the number of cases with a high content of neoplastic cells would probably have increased and hence the probability of high score predications. In spite of this, we got a relatively high proportion of tumours with high‐confident scores. Despite a study period of 4 years, there were, for some tumour types, few samples in each subgroup, which may affect the interpretation of the results. Overall, it is crucial to interpret the results from the methylation profiling in the context of clinical, radiological and histopathological information.

In conclusion, our national population‐based study shows that DNA methylation classification is of value for all types of CNS tumours and has an important role also in tumours with a low tumour cell content. DNA methylation can enhance the diagnostic information and potentially help identify rare entities. We find DNA methylation‐based classification to be an invaluable tool in the diagnostics of paediatric CNS tumours and advocate its integration in real time standard diagnostics.

## CONFLICT OF INTEREST

SMP declare patent PCT/EP2016/055337 DNA‐methylation‐based method for classifying tumour species. All other authors have no conflicts of interest to report.

## ETHICS STATEMENT

The study was approved by the regional ethics committee in Gothenburg, Sweden (Dnr 604‐12, T1162‐16). Informed consent was obtained from the guardians.

## AUTHOR CONTRIBUTIONS

ES, MS, BL and HC designed the study and ES and HC coordinated it. ML performed most of the experiments together with AW, SFV and AD. TP did the neuropathological re‐evaluation. SMP contributed with methylation algorithms, and algorithms were run by HC, ML and TK. TOB and SD evaluated the histological tumour cell content. MS, ES, SH, AÖ, PN, MEH and P‐ES referred patients to the study. ES, TK and SFV prepared figures, and ES wrote the manuscript with input from all other authors. All authors read and approved the final manuscript.

## Supporting information


**Figure S1.** Histopathology for two cases with revised diagnosis. A) Case 10 (Table 2) a revised histopathology diagnosis of epithelioid glioblastoma with positive staining for Vimentin and Olig2. Tumour cells show loss of p16, absence of GFAP and lack of reticulin fibres. CD34 is only positive in endothelial cells. Positive Ki67 marker in 5–10% of the tumour cells. B) Case 12 (Table 2) with the revised diagnosis of metastatic medulloblastoma with expression of synaptophysin and OTX2. Vimentin is negative.

**Figure S2.** Result of DNA methylation classification of paediatric CNS tumours using MNP version 12.5. Of the 240 profiled cases, 208 tumours (87%) were classified with a high calibrated score, ≥0.84.

**Table S1:** Classification of medulloblastoma group 3 and 4 samples using different versions of the DNA methylation‐based classifier.

## Data Availability

The data that support the findings of this study are available on request from the corresponding author. The data are not publicly available due to privacy or ethical restrictions.
