# Supplementary figures and images for: DNA methylation profiling improves routine diagnosis of paediatric central nervous system tumours: A prospective population‐based study
Source: Neuropathol Appl Neurobiol. 2022 Aug 3;48(6):e12838. doi: 10.1111/nan.12838 (PMC9543790; doi:10.1111/nan.12838)

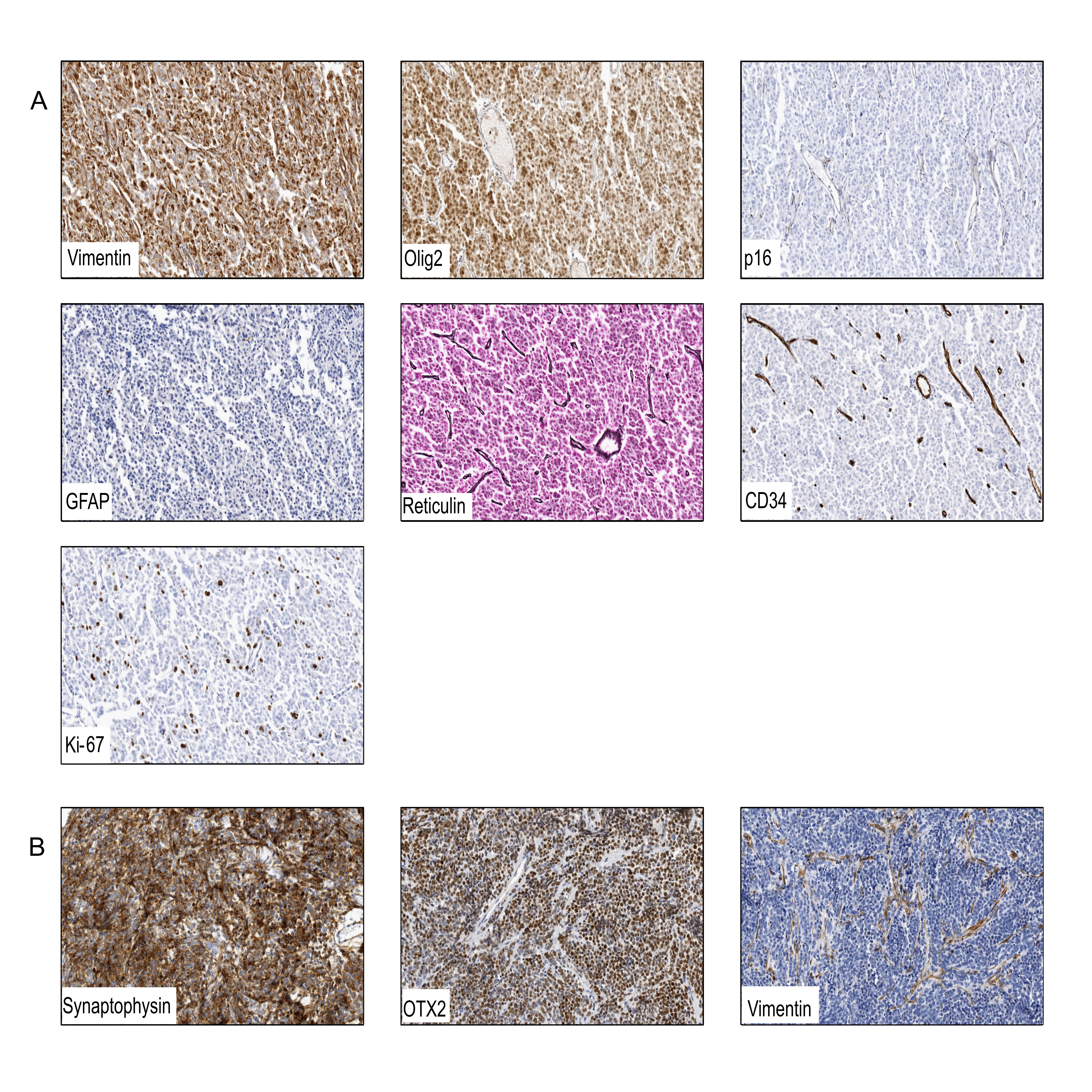

Supplement: Supplementary file 1 — Figure S1. Histopathology for two cases with revised diagnosis. A) Case 10 (Table 2) a revised histopathology diagnosis of epithelioid glioblastoma with positive staining for Vimentin and Olig2. Tumour cells show loss of p16, absence of GFAP and lack of reticulin fibres. CD34 is only positive in endothelial cells. Positive Ki67 marker in 5–10% of the tumour cells. B) Case 12 (Table 2) with the revised diagnosis of metastatic medulloblastoma with expression of synaptophysin and OTX2. Vimentin is negative. [file NAN-48-0-s001.tiff]

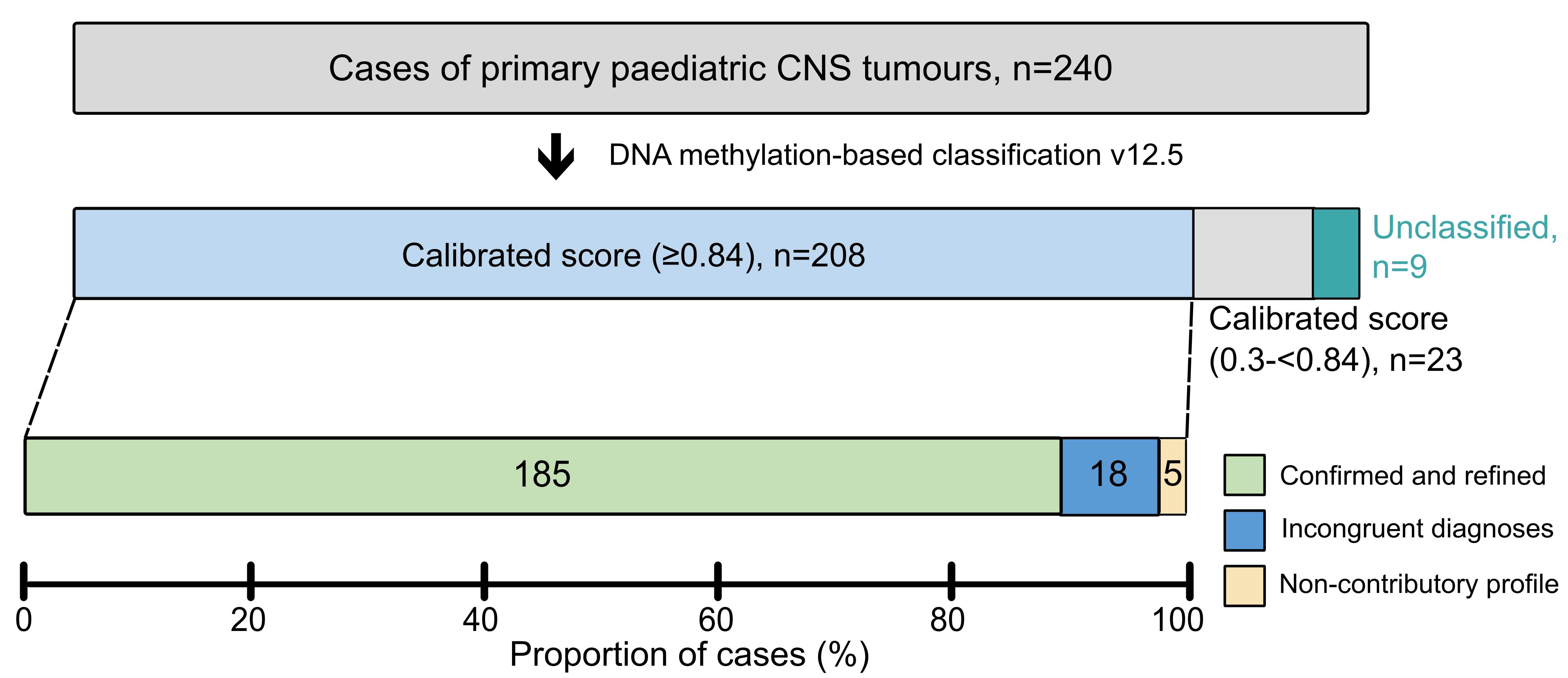

Supplement: Supplementary file 2 — Figure S2. Result of DNA methylation classification of paediatric CNS tumours using MNP version 12.5. Of the 240 profiled cases, 208 tumours (87%) were classified with a high calibrated score, ≥0.84. [file NAN-48-0-s002.tif]
