# Supplementary material for: DNA methylation profiling improves routine diagnosis of paediatric central nervous system tumours: A prospective population‐based study
Source: Neuropathol Appl Neurobiol. 2022 Aug 3;48(6):e12838. doi: 10.1111/nan.12838 (PMC9543790; doi:10.1111/nan.12838)
Supplement: Supplementary file 3 — Table S1: Classification of medulloblastoma group 3 and 4 samples using different versions of the DNA methylation‐based classifier. [file NAN-48-0-s003.pdf]

Sample-ID	v11.4		v12.5								MB classifier 1.0	
	Prediction	Max score	prediction superfamily	maxscore superfamily	prediction family	maxscore family	prediction class	maxscore class	prediction subclass	maxscore subclass	prediction	maxscore
1	MB, subclass group 3	0.990	MB non-WNT/non-SHH activated	0.999	MB non-WNT/non-SHH activated	0.999	MB Group 3	0.997	MB Group 3, subclass III	0.781	III	0.945
2	MB, subclass group 4	0.990	MB non-WNT/non-SHH activated	1.000	MB non-WNT/non-SHH activated	1.000	MB Group 4	1.000	MB Group 4, subclass VI	0.994	VI	0.997
3	MB, subclass group 4	0.900	MB non-WNT/non-SHH activated	1.000	MB non-WNT/non-SHH activated	1.000	MB Group 4	1.000	MB Group 4, subclass V	1.000	V	1.000
4	MB, subclass group 4	0.960	MB non-WNT/non-SHH activated	1.000	MB non-WNT/non-SHH activated	1.000	MB Group 4	0.958	MB Group 4, subclass V	0.956	V	0.996
5	MB, subclass group 4	0.960	MB non-WNT/non-SHH activated	1.000	MB non-WNT/non-SHH activated	1.000	MB Group 4	0.999	MB Group 4, subclass V	0.997	V	0.981
6	MB, subclass group 4	0.990	MB non-WNT/non-SHH activated	1.000	MB non-WNT/non-SHH activated	1.000	MB Group 4	1.000	MB Group 4, subclass VIII	1.000	VIII	0.999
7	MB, subclass group 4	0.990	MB non-WNT/non-SHH activated	0.999	MB non-WNT/non-SHH activated	0.999	MB Group 4	0.997	MB Group 4, subclass VI	0.802	VI	0.979
8	MB, subclass group 4	0.990	MB non-WNT/non-SHH activated	0.997	MB non-WNT/non-SHH activated	0.997	MB Group 4	0.996	MB Group 4, subclass VIII	0.650	VI	0.795
9	MB, subclass group 4	0.990	MB non-WNT/non-SHH activated	0.999	MB non-WNT/non-SHH activated	0.999	MB Group 4	0.999	MB Group 4, subclass VIII	0.997	VIII	0.999
10	MB, subclass group 4	0.990	MB non-WNT/non-SHH activated	0.998	MB non-WNT/non-SHH activated	0.998	MB Group 4	0.998	MB Group 4, subclass VIII	0.796	VI	0.583
11	MB, subclass group 4	0.990	MB non-WNT/non-SHH activated	0.999	MB non-WNT/non-SHH activated	0.999	MB Group 4	0.990	MB Group 4, subclass VII	0.970	VII	0.992
12	MB, subclass group 4	0.990	MB non-WNT/non-SHH activated	1.000	MB non-WNT/non-SHH activated	1.000	MB Group 4	1.000	MB Group 4, subclass VIII	1.000	VIII	1.000
13	MB, subclass group 3	0.990	MB non-WNT/non-SHH activated	0.999	MB non-WNT/non-SHH activated	0.999	MB Group 3	0.999	MB Group 3, subclass II	0.993	II	0.944
14	MB, subclass group 4	0.990	MB non-WNT/non-SHH activated	1.000	MB non-WNT/non-SHH activated	1.000	MB Group 4	1.000	MB Group 4, subclass V	1.000	V	1.000
15	MB, subclass group 4	0.990	MB non-WNT/non-SHH activated	1.000	MB non-WNT/non-SHH activated	1.000	MB Group 4	0.999	MB Group 4, subclass VI	0.818	VI	0.861
16	MB, subclass group 3	0.990	MB non-WNT/non-SHH activated	1.000	MB non-WNT/non-SHH activated	1.000	MB Group 3	1.000	MB Group 3, subclass II	1.000	II	0.998
17	MB, subclass group 4	0.990	MB non-WNT/non-SHH activated	1.000	MB non-WNT/non-SHH activated	1.000	MB Group 4	1.000	MB Group 4, subclass VI	1.000	VI	0.998
18	MB, subclass group 3	0.990	MB non-WNT/non-SHH activated	1.000	MB non-WNT/non-SHH activated	1.000	MB Group 3	1.000	MB Group 3, subclass IV	0.999	IV	0.997
19	MB, subclass group 4	0.990	MB non-WNT/non-SHH activated	1.000	MB non-WNT/non-SHH activated	1.000	MB Group 4	1.000	MB Group 4, subclass VIII	1.000	VIII	0.999
20	MB, subclass group 4	0.990	MB non-WNT/non-SHH activated	1.000	MB non-WNT/non-SHH activated	1.000	MB Group 4	1.000	MB Group 4, subclass VI	0.999	VI	0.995
21	MB, subclass group 4	0.990	MB non-WNT/non-SHH activated	1.000	MB non-WNT/non-SHH activated	1.000	MB Group 4	1.000	MB Group 4, subclass VII	1.000	VII	1.000
22	MB, subclass group 4	0.990	MB non-WNT/non-SHH activated	1.000	MB non-WNT/non-SHH activated	1.000	MB Group 4	1.000	MB Group 4, subclass VIII	1.000	VIII	0.999
23	MB, subclass group 4	0.860	MB non-WNT/non-SHH activated	0.999	MB non-WNT/non-SHH activated	0.999	MB Group 4	0.997	MB Group 4, subclass VII	0.996	VII	0.997
24	MB, subclass group 4	0.990	MB non-WNT/non-SHH activated	0.999	MB non-WNT/non-SHH activated	0.999	MB Group 4	0.999	MB Group 4, subclass VI	0.959	VI	0.979
25	MB, subclass group 4	0.990	MB non-WNT/non-SHH activated	1.000	MB non-WNT/non-SHH activated	1.000	MB Group 4	1.000	MB Group 4, subclass VIII	1.000	VIII	0.999
26	MB, subclass group 4	0.990	MB non-WNT/non-SHH activated	1.000	MB non-WNT/non-SHH activated	1.000	MB Group 4	1.000	MB Group 4, subclass VI	0.770	VI	0.930

MB; medulloblastoma
